# Ecological Traits and Trophic Plasticity in The Greater Pipefish *Syngnathus acus* in the NW Iberian Peninsula

**DOI:** 10.3390/biology11050712

**Published:** 2022-05-07

**Authors:** Miquel Planas

**Affiliations:** Department of Marine Ecology and Resources, Instituto de Investigaciones Marinas (CSIC), 36208 Vigo, Spain; mplanas@iim.csic.es; Tel.: +34-986214457

**Keywords:** pipefish, *Syngnathus acus*, biology, ecology, reproduction, trophic plasticity, stable isotopes, diet

## Abstract

**Simple Summary:**

The population of the pipefish *Syngnathus acus* inhabiting Cíes Archipelago (NW Spain) was monitored in 2017–2018 for spatial and temporal changes in abundances, reproduction traits, trophic niche occupancy, and dietary regimes across reproduction states, through an isotopic (δ^13^C and δ^15^N) approach. The population consisted almost exclusively of large adults, whose abundances decreased significantly from mid-autumn after the breeding season. *S. acus* is a secondary consumer that prefers amphipods, but mature specimens were less selective than immature fish. The present study highlights the outstanding size of the fish and the exceptional occurrence of breeders on the studied area.

**Abstract:**

The great pipefish *Syngnathus acus* is one of the most representative European syngnathids, being highly associated with seagrass and macroalgal beds. Surprisingly, the ecology of this large ovoviviparous marine fish has received scanty attention. The population inhabiting three sites on Cíes Archipelago (Atlantic Islands National Park, NW Spain) was monitored in 2017–2018 for spatial and temporal changes in abundances, reproduction traits, trophic niche occupancy, and dietary regimes across reproduction states, through an isotopic (δ^13^C and δ^15^N) approach. Abundances were highly variable across seasons and sites, decreasing significantly from mid-autumn. The population consisted almost exclusively of large adults that migrate by the end of the breeding season, which extended from mid-spring to summer. Operational sex ratios suggest that the species is sex-role reversed. *S. acus* is a secondary consumer (Trophic position = 3.36 ± 0.05), preferring amphipods but displaying annual and seasonal dietary plasticity. Mature fish were less selective than immatures (especially females), with a higher preference for amphipods (36–68%) in the former. The second most-preferred prey were carideans, copepods, or isopods, depending on the year and the reproduction state. Overall, the wider trophic niches in females and immature specimens compared to males and mature fish would indicate a higher variability in both the use of prey resources and/or their origin. The present study highlights the trophic plasticity and unique features of *S. acus* population in the Cíes Archipelago, especially regarding the outstanding size of the fish and the exceptional occurrence of breeders.

## 1. Introduction

The Family Syngnathidae includes a large and diverse group of vulnerable and cryptic fishes that are characterized by singular morphological and biological features, a tiny mouth at the end of a tubular snout, and male parental care. Pipefishes are mostly distributed in shallow waters on the coasts of temperate and tropical regions. In *Syngnathus* pipefishes, the eggs and developing embryos are enclosed within specialized brooding structures (i.e., marsupia with protective pouch flaps) located on the ventral side of the trunk or tail. The greater pipefish, *Syngnathus acus* Linnaeus, 1758 is a demersal syngnathid inhabiting brackish marine areas on the Mediterranean, Aegean, and Black seas, and the Eastern Atlantic [1]. *S. acus* is an ovoviviparous tail-brooding species that generally reproduces three times each year [2]. It is currently listed as Least Concern by the IUCN Red List [3]. 

Despite its common occurrence in brackish seaweeds and seagrass beds, the biology and ecology of *S. acus* have been poorly investigated, and data availability is limited to a few studies [4,5,6,7,8] (among others). The reproduction of the species [2] and its feeding habits and prey preference [9,10,11] were investigated in the Aegean Sea; Jennings and van der Molen [12] estimated the trophic position in a few specimens from the Celtic Sea and the English Channel, whereas Silva et al. [13] focused on its development and early life history in ex-situ experiments.

The distribution, the habitat preference, and some trophic features in syngnathids inhabiting Cíes Archipelago, on the Atlantic Islands National Park (NW Spain), have been recently assessed, focusing on three sympatric species: the long-snouted seahorse *Hippocampus guttulatus*, the snake pipefish *Entelurus aequoreus*, and the great pipefish *Syngnathus acus* [14,15]. Among them, the latter was the most representative species, with a wider distribution, preferentially on macroalgal beds [16].

The available data on syngnathid populations in Cíes Archipelago indicate high seasonal and spatial variability in distribution and abundances [14,15]. Such variability seemed to be governed by seasonal changes in temperature, the extension and integrity of macroalgal beds, the structure of epifauna assemblages, and potential migratory events. These factors might also affect resource availability, resulting in potential dietary shifts in syngnathids, which commonly feed on small crustaceans [17] but might show some plasticity in prey selection, depending on the species [18,19].

Some of the most interesting topics in marine ecology are the assessment of the trophic web and the inference of feeding regimes of targeted species. In Cíes Archipelago, the former was investigated via an isotopic approach, concluding that the three syngnathids occurring in the area were rather similar in trophic features [14]. Isotopic profiles in animals vary depending on the trophic level occupied, and the dietary regime [20,21,22,23,24,25,26]. Stable isotope analysis (SIA) is a powerful tool for inferring temporal and spatial changes in feeding regimes and migrations [27,28], or to assess the parental contribution of dietary nutrients into eggs/offspring [29,30,31,32,33].

The importance of *S. acus* as targeted species, especially in Cíes Archipelago, is based on the following traits: (a) It is the largest and most representative syngnathid on the Eastern European coasts; (b) it is the most abundant syngnathid in Cíes Archipelago; (c) it is a migratory species that is highly associated with macroalgae assemblages; (d) its trophic niche is highly similar to other much less abundant syngnathids co-occurring in Cíes Archipelago; and (e) studies on the global assessment of *S. acus* populations are almost lacking. In the present study, the ecological traits and the variability in the feeding regime of *S. acus* inhabiting Cíes Archipelago were investigated in detail, with the aim of (a) improving knowledge on the biology, population structure, and breeding features; (b) inferring temporal and spatial changes in the feeding regime, using stable isotope profiles; and (c) assessing the relationship between diet and maturity state in males and females.

## 2. Materials and Methods

### 2.1. Study Sites

**Figure 1:**
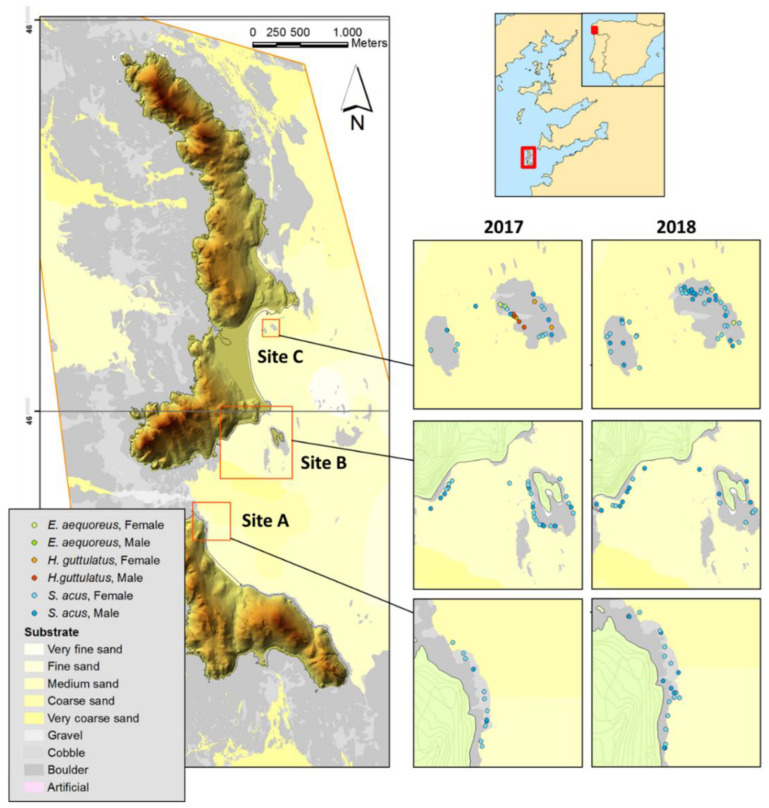
Sites (A, B, and C) surveyed for *Syngnathus acus* monitoring in Cíes Archipelago (Galicia, NW Iberian Peninsula) in spring, summer, and autumn (2017–2018). Substrate characteristics and syngnathids captured (*S. acus*, *Entelurus aequoreus*, and *Hippocampus guttulatus*) are shown.

The study was conducted in Cíes Archipelago (42°13′ N, 8°54′ W) as part of the Atlantic Islands Marine National Park (NW Iberian Peninsula) (Figure 1). The Archipelago was declared a Natural Park, Special Protection Area (SPA), Site of Community Importance (SCI), OSPAR area, and UNESCO World Heritage candidate [34]. Further information on the characteristics of Cíes Archipelago and its biotopes are provided by Fernández et al. [35].

**Figure 2:**
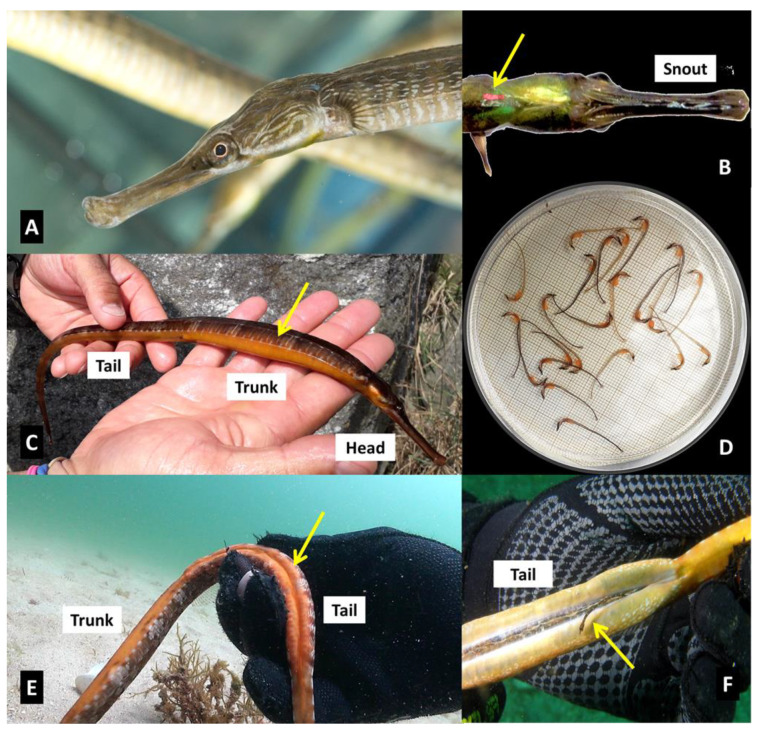
*Syngnathus acus*. **A**—Head morphology; **B**—Specimen marked with fluorescent VIFE; **C**—Ovigerous female showing a swollen trunk; **D**—Near-term embryos with small yolk-sacs; **E**—Pregnant male showing the enclosed breeding pouch below the tail; **F**—A newborn emerging from the brooding structure.

Based on previous knowledge (i.e., seaweed cover, substrate characteristics, exposure level to open water) [14,15,16], three subtidal sites (A, B, and C) (Figure 1) were selected along the east coast of Cíes Archipelago, located on the outer area of the Ría de Vigo. The sites were positioned near the coastline (2–15 m depth) on rocky bottoms frequently interrupted by sandy patches, and visited in spring, summer, and autumn in 2017 and 2018 (two visual censuses per site and season). Two pairs of divers conducted 36 diurnal standard underwater visual censuses (UVC) (50 min per survey; about 144 diving hours). The total areas explored per survey at sites A, B, and C were 2450, 5625, and 2153 m^2^, respectively. Sighted specimens of the greater pipefish *Syngnathus acus* Linnaeus, 1758 (Family Syngnathidae) (Figure 2) were recorded and captured by the divers searching adjacently (belt transects) and separated by the maximum distance allowed for horizontal visibility (usually 2.5 m). The depth and location of fish capture were annotated.

### 2.2. Fish Collection and Sampling

*Syngnathus acus* specimens (Figure 2A) were hand-caught collected, introduced in numbered plastic bags, and transferred to a support boat. Subsequently, the fish were anaesthetized with a solution of Ethyl 3-aminobenzoate methane sulfonate (MS-222; 0.1 g L^−1^; Sigma-Aldrich Co., St. Louis, MI, USA), morphologically identified, weighted (W, g), sized for standard length (SL, cm), and marked subcutaneously using visible implant fluorescent elastomers (VIFE; Northwest Marine Technology Inc., Anacortes, WA, USA) (Figure 2B). Dorsal fin samples were taken via fin-clipping [36,37], transferred to screw-capped tubes containing 95% ethanol, and conserved at 4 °C for further genetic and stable isotope analysis (SIA). The presence of previous marks (recapture events), sex, reproductive condition, meristics (fin rays, body rings), and body coloration was also annotated whenever possible. All fishes were released at the capture site within 2–3 h after sampling.

Simultaneously to fish monitoring, samples of potential prey for *S. acus* were collected in the same sampling sites of this study for further identification and stable isotope analysis [14].

The morphological identifications of the captured fish were confirmed genetically [38], using the marker cytochrome b (Cytb;1149 base pairs) [39,40] in DNA extracted from dorsal fin samples.

The operational sex ratio (OSR) at a given time was calculated as the number of males available for mating as a proportion of all adults available for mating [41]. The reproductive condition was recorded, considering the trunk shape in females (i.e., full gonads with hydrated eggs) (Figure 2C) and pregnancy in males (i.e., fully enclosed brood pouches carrying fertilized eggs/embryos) (Figure 2D–F).

To minimize hampering effects in fishes, morphological analysis and sampling of developing eggs/larvae in brood pouches were limited to a few males. Sampling for size measurement and stable isotopes included fertilized eggs at stage B (cleavage stage; *n* = 1 batch), embryos at stages H (eyes pigmented; *n* = 9) and I (larva/prerelease stage; reduced yolk-sac; *n* = 5), and newly released juveniles (stage J; yolk-sacs almost fully resorbed; snout formation completed; *n* = 4) [42]. Stage H would correspond to larvae that were newly emerged from the egg, but retained within the brood pouch, with jaw formation having commenced and eyes being partially pigmented [43].

The weight–length relationship in adults was investigated using the following equation:

W = a SL ^b^ (1)

where W is the body wet weight, SL is the standard length, a is an empirical coefficient, and b is the allometric exponent. For the standard length (SL) measurement, the fishes were measured directly on a plate, including a measurement scale.

Daily weight-specific growth rates (G; % day^−1)^ were estimated from the weight data in re-sighted individuals:

G = 100 (e^g^−1) (2)

where the instantaneous growth coefficient g was:

g = (ln W_2_−Ln W_1_)/(t_2_−t_1_) (3)

where W_2_ and W_1_ are fish wet weights (g) on days t_2_ (recapture) and t_1_ (first capture), respectively.

### 2.3. Stable Isotopes Analysis (SIA)

For δ^13^C, δ^15^N, total C, and total N analyses, the samples were rinsed with distilled water, transferred to tin capsules, dried in an oven at 60 °C for 24 h, and weighted (±1 μg). The samples were analyzed at SAI (University of A Coruña) by continuous-flow isotope ratio mass spectrometry using a FlashEA1112 elemental analyzer (Thermo Finnigan, Italy) coupled to a Delta Plus mass spectrometer (FinniganMat, Germany) through a Conflo II interface. Isotopic values are expressed as permil (‰) in conventional delta relative to VPDB (Vienna Pee Dee Belemnite) and atmospheric air. As part of an analytical batch run, a set of international reference materials for δ^15^N values (IAEA-N-1, IAEA-N-2, and USGS25) and δ^13^C values (NBS 22, IAEA-CH-6, and USGS24) were analyzed. The range of C:N ratios in fin tissues (2.8–3.5) were within the range in the reference materials (0.4–6.9) used. The precision (standard deviation) for the analysis of δ^13^C and δ^15^N of the laboratory standard (acetanilide) was ±0.15‰ (1-sigma, *n* = 10). Standards were run every 10 biological samples. The isotopic analysis procedure fulfils the requirements of the ISO 9001 standard. The laboratory is submitted to annual intercalibration exercises (e.g., Forensic isotope ratio mass spectrometry scheme—FIRMS, LGC Standards, UK).

Due to the low lipid content in the fin samples conserved in ethanol (<5% lipids, C:N < 3.56) [44,45], normalization for lipid correction in the fin samples was not necessary [37,46]. However, the C:N values in the epifauna indicated that the lipid content was higher than 5%, and specific conversion factors constructed for lipid normalization were applied [14]. Additionally, some epifaunal groups were submitted to acidification before SIA [14] by adding dilute (10%) HCl drop-by-drop, until CO_2_ release was no longer observed [47,48].

### 2.4. Data Analysis

Data and statistical analyses were conducted in R v.3.6.1 [49]. All means are reported with standard deviations. A P-value threshold of 0.05 was considered significant in all statistical analyses.

Data comparisons across the groups were performed using non-parametrical tests (Kruskal–Wallis) [50]. Significant differences between the groups were pairwise compared using the Wilcoxon test (p. adj = Bonferroni) (Pgirmess v1.6.9 package) [51]. The analyses included comparisons for abundances, standard length, wet weight, and δ^13^C and δ^15^N across the seasons (spring, summer, and autumn) and reproductive stages (ovigerous females, pregnant males, nonovigerous females, and non-pregnant males).

Two-dimensional non-metric multidimensional scaling (NMDS; Euclidean distances) plots on the variation in *S. acus* abundances were constructed, considering the years of the survey (2017 and 2018) and the seasons (Sp—spring; Su—summer; Au—autumn). NMDS is based on a Bray–Curtis (BC) dissimilarity matrix [52], and it was performed with the package vegan v.2.5-7 [53].

Principal Component Analyses (PCA), including fish wet weight and isotopic data as variables, were performed for year, sex, and reproductive states, using factoMineR v2.3 [54], factoextra v1.0.7 [55], and corrplot v0.8.4 [56] packages. The data values were standardized (mean = 0; SD = 1) for clustering and PCA.

Fish trophic position (TP) was estimated from the δ^15^N values using tRophicPosition v. 0.7.7 [57], an R package incorporating a Bayesian model for the calculation of consumer TP at the population level. The bivalve *Musculus costulatus* was sampled simultaneously to fishes, and the average seasonal levels were used as a trophic baseline (TP = 2) [14,58]. Isotopic values for *M. costulatus* were −17.71‰ ± 0.07 for δ^13^C and 5.32 ± 0.26‰ for δ^15^N. An experimentally derived TDF value (3.9 for δ^15^N) for syngnathids was applied [58]. Comparisons between the groups were assessed through a t-test.

Niche regions and pairwise niche overlap in fishes were assessed using a δ^13^C and δ^15^N bi-plot as multidimensional niche indicator data. The niche region was defined as the joint probability density function of the multidimensional niche indicators, at a probability alpha of 95%. Uncertainty was accounted for in a Bayesian framework. The analysis provides directional estimates of niche overlap, accounts for species-specific distributions in the multivariate niche space, and produces unique and consistent bivariate projections of the multivariate niche region [59]. The packages SIBER v.2.1.4 [60] and NicheRover v.1.1.0 [61] were used to assess differences in the trophic niche features. The total convex hull areas (TA) and core trophic niche breadths were estimated using SIBER (Stable Isotope Bayesian Ellipses), while correcting for variable sample sizes (SEAc). The total trophic overlap values for 95% TA were estimated using nicheROVER, a method that is insensitive to sample size and incorporates statistical uncertainty using Bayesian methods [59].

From the isotopic profiles in selected potential prey (amphipods, harpacticoid copepods, carideans, isopods, and mysidaceans) (see [58] for isotopic data) and in the consumer *S. acus*, the dietary regimes across the years and reproductive stages were estimated using Bayesian Mixing Models (SIMM) [62], using the MixSIAR package v.3.1.12 [63]. The assessment of the dietary regime in the pipefishes was performed, accounting for isotopic variability in the prey across the years, seasons, and sites [14]. The SIMM procedure is fully described in [58]. In short, SIMM polygons were constructed with isotopic profiles adjusted for TDFs, to determine the proportion of consumers that were included inside the mixing polygon bound by all potential sources [60,64,65]. TDF values applied were experimentally derived for these fishes (2.5 for δ^13^C; 3.9 for δ^15^N) [46,58]. Two individuals with a low probability (<5%) of being positioned inside the mixing polygon were not included in the subsequent Bayesian models [65]. The models were run with Markov chain Monte Carlo (MCMC) parameters of three chains of 1,000,000 iterations, and a burn-in phase of 500,000 (very long run). The model included individuals as a random effect, and one error term (process error). Convergence and diagnostic statistics were evaluated using both Gelman–Rubin (variables < 1.05) and Geweke (number of variables outside ± 1.96 in each chain) tests. Bayesian model outputs are reported as mean ± 95% CI.

The graphics were constructed using Excel 2016, ggplot2 v3.3.0 [66], and lattice v0.20–41 [67] packages.

### 2.5. Ethics

Fish capture, handling, and sampling were conducted in compliance with all bioethics standards on animal experimentation of the Spanish Government (R.D. 1201/2005, 10 October) and the Regional Government Xunta de Galicia (Reference REGA ES360570202001/16/FUN/BIOL.AN/MPO02).

## 3. Results

### 3.1. Abundances and Population Characteristics

**Figure 3:**
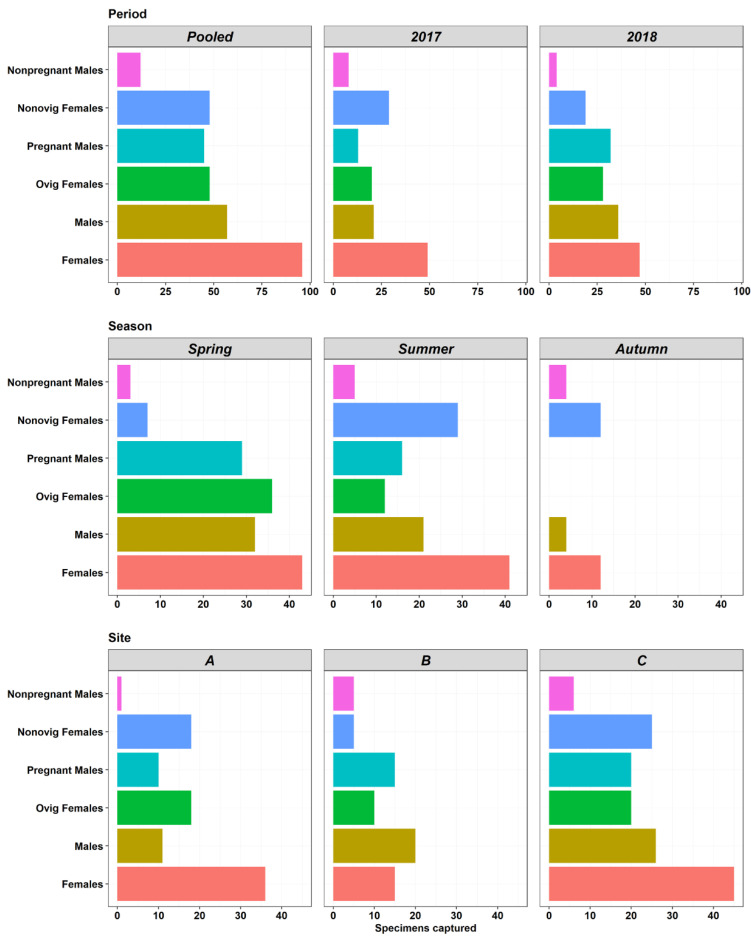
*Syngnathus acus* specimens captured in surveys conducted in spring, summer, and autumn (2017 and 2018) at sites A, B, and C, in Cíes Archipelago.

A total of 153 *Syngnathus acus* specimens were captured (Table 1, Figure 3) (70 in 2017 and 83 in 2018). The most common meristic characteristics of the pipefishes were: 62 body rings (range: 61–64), 20 trunk rings (19–20), 42 tail rings (41–44), 12 pectoral-fin rays (9–12), 38 dorsal-fin rays (37–41), 3 anal-fin rays, and 10 caudal-fin rays.

The total captures at sites A, B, and C accounted for 22, 31, and 47% of the total specimens, respectively, but the differences were not significant, due to large deviations (K-W; Χ^2^_(3)_ = 4.99; *p* = 0.082) (Figure S1). The average abundance was low (12.5 fish km^−^), corresponding to 3.9, 15.2, and 18.3 fish km^−^ from sites A, B, and C, respectively. The highest abundance was recorded in the summer of 2018 at site C (55.3 fish km^−^). The most fish were collected in spring (49%), and to a lesser extent, in summer (41%). Captures in autumn were sharply reduced (10%), especially in 2018 (2.6%). A graphical summary of abundances (nMDS plots) across the years, seasons, and sites are provided in Figure S2. Females (63%) outnumbered males (37%), and the captures significantly differed across seasons (Kruskal–Wallis; Χ^2^_(3)_ = 14.7; *p* < 0.0001) (Figure S1).

The average size and weight values were 34.4 ± 6.8 cm SL (range: 16.4–49.8 cm) and 33.3 ± 19.3 g (range: 2.8–102.7 g), respectively (Table 2 and Table S1). Adult specimens larger than 30 cm SL accounted for 78% of the total captures.

A total of 11 *S. acus* individuals (six females and five males) were re-sighted, most of them in 2018. Recaptures generally occurred in the season following the first capture, but two individuals caught in 2017 were recaptured in 2018. In most cases, the first capture and further re-sighting occurred at the same site (three in site B and five in site C). Two individuals initially marked in site C were recaptured in site B, at a distance of about 1 km. Another specimen previously captured in site B was re-sighted in site C. Despite the low number of individuals re-sighted, daily weight-specific growth rates (G) were roughly estimated in eight specimens, ranging from 0.08% day^−1^ (large individuals) to 0.43% day^−1^ (small individuals) (Figure S3).

Length–weight relationships for the whole population (Figure S4) indicated positive allometry (b = 3.20), mostly due to the high values of coefficient b in immature individuals (b = 3.32–3.35) compared to mature fishes (b = 2.97–2.83).

### 3.2. Reproduction Traits

Mature females and males were mostly captured in spring, and to a lesser extent, in summer (Table 1, Figure 3). Opposite to spring, the relative occurrence of pregnant males in summer was higher than for ovigerous females. Average sex ratios (males:males+females) in 2017 and 2018 were 0.30 and 0.43, respectively, but these differed between sites, with a higher relative occurrence of females in sites A and C (Figure 3). The sex ratios in spring, summer, and autumn were 0.43, 0.34, and 0.25, respectively. The operational sex ratios (OSR; Table 1) for the breeding period (spring and summer) were highly variable across the sites, seasons, and years, but the population was slightly female-biased (0.45) in spring, the peak of the breeding season (0.33 in spring of 2017; 0.54 in spring of 2018). In summer, the breeding population was slightly male-biased (0.57; 0.71 in 2017; 0.52 in 2018).

Nonovigerous females (31.8 ± 6.8 cm; range: 19.5–45.0 cm) were significantly smaller than ovigerous females (36.2 ± 5.6 cm; range: 19.1–47.4 cm) and pregnant males (36.3 ± 3.2 cm; range: 24.0–49.8 cm) (Table 2 and Table S1, Figure S5). Half of the total females displayed signs of maturity, with swollen trunks and brilliant coloration.

The eggs and developing larvae were aligned inside the marsupium, forming four strings that generally occupied the entire brooding structure. Morphologically, all of the eggs/embryos in a male’s brood pouch were frequently at the same development stage. However, a few males carried more than one partial clutch, or showed partially filled pouches. The eggs contained within a brooding structure were counted in one pregnant male, accounting for 166 eggs, which covered about 90% of the total available surface. The average egg diameter in stage B was 2.03 ± 0.19 mm. The lengths in pre-larvae and larvae in stages H, I, and J were 2.18 ± 0.49, 2.79 ± 0.18, and 3.02 ± 0.07 cm, respectively (Table 3). 

### 3.3. Isotopic Profiles

The average isotopic values for the whole population were −15.4 ± 0.5‰ for δ^13^C, and 11.1 ± 0.6‰ for δ^15^N (Table S2). Females and males displayed similar δ^13^C values (−15.5–15.3‰) (K-W; Χ^2^_(3)_ = 4.22; *p* = 0.240), but differed significantly in δ^15^N across the maturity stages (10.7–11.4‰) (K-W; Χ^2^_(3)_ = 8.11; *p* = 0.044) (Table 2, Figure S5). The average values for δ^15^N in ovigerous females and pregnant males were similar (11.3–11.4 ‰) (*p* = 0.757), and significantly higher than in immature specimens (10.7–10.9‰) (*p* < 0.05).

**Figure 4:**
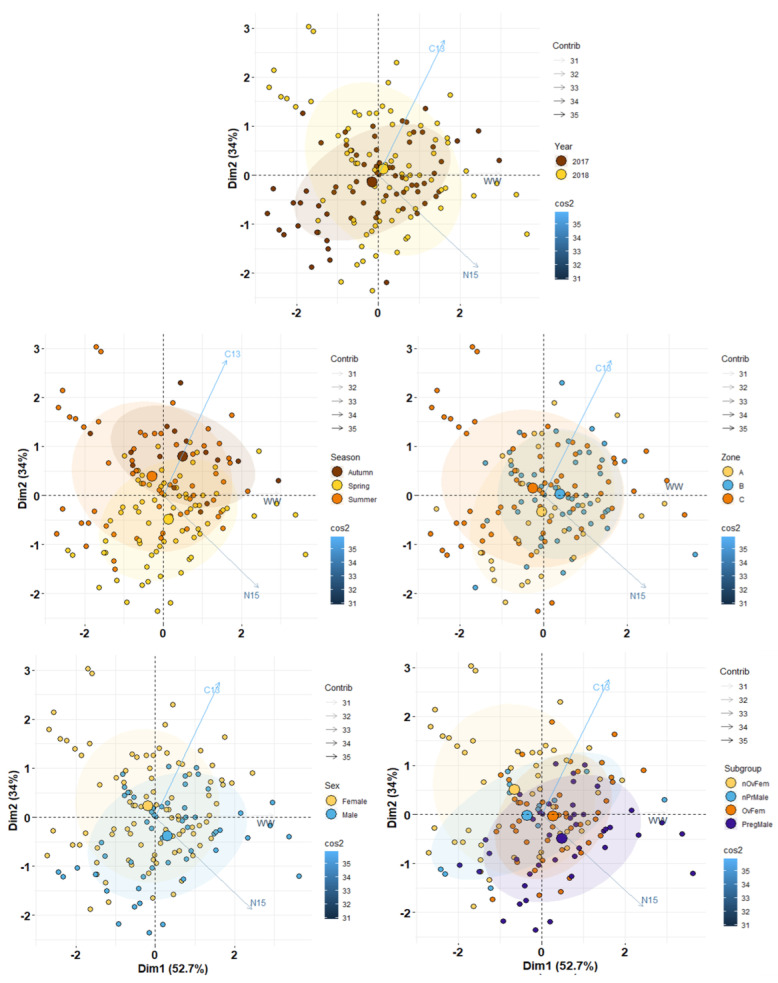
Factor score plots for the PCA on *S. acus* collected in spring, summer, and autumn (2017 and 2018) on sites A, B, and C in Cíes Archipelago. Variables: WW—Wet weight, C13—δ^13^C (absolute values) and N15—δ^15^N. Ellipses correspond to 95% confidence. Plots provided for year, season, site, sex, and reproductive status (Nonovigerous and ovigerous females, non-pregnant and pregnant males).

The principal component analysis (PCA) performed on the weight and isotopic data revealed that the first two factors explained as much as 86.7% of the total (Figure 4). The centroids for year and the surveyed sites were very close to each other, indicating relative isotopic stability regarding those variables. However, data dispersion was higher in samples from 2017 and at site C. This finding was mainly caused by lower δ^15^N values in non-ovigerous females. Regarding seasonal changes, the transition from spring to autumn was characterized by a progressive δ^13^C decrease. The PCA plots for sex and maturity state denoted higher δ^15^N values in males, and an alignment of centroids following increasing δ^15^N values from immature females to pregnant males. In contrast, the contribution of δ^13^C values on the centroid positions was almost negligible. 

### 3.4. Isotopic Inheritance

The isotopic profiles in embryos and newly released juveniles were rather similar, especially for δ^15^N (Table 3). Isotopic values in B-stage eggs were −16.6‰ for δ^13^C and 10.9‰ for δ^15^N. The values at that stage were slightly lower (1.5‰ for δ^13^C and 1.1‰ for δ^15^N) than in the corresponding males. The most advanced developmental stages (H to J) were slightly enriched compared to the B stage, ranging from −16.3‰ to −15.2‰ for δ^13^C, and from 11.5‰ to 11.8‰ for δ^15^N. Furthermore, there was a direct linear relationship between the isotopic values in stages H–J and those in brooding males, so that the isotopic ratios between pre-larva/larva and males ranged from 0.97 to 1.05 for δ^13^C, and from 0.99 to 1.04 for δ^15^N, and did not differ from 1 (*p* = 0.525 for δ^13^C; *p* = 0.415 for δ^15^N).

### 3.5. Trophic Position

The average trophic position (TP) of *S. acus* was 3.36 ± 0.05 (Table 4). TP values for the males and females differed significantly (3.39 ± 0.05 and 3.33 ± 0.05, respectively) (*t*-test, t = 7.654, *n* = 77, *p* < 0.0001), whereas mature individuals occupied a higher trophic position than immature fishes (3.41 ± 0.05 and 3.26 ± 0.05, respectively) (t = 16.488, *n* = 132, *p* < 0.0001). TP values in individuals collected in summer were significantly lower than in those from the spring (8.5% decrease; t = 20.365, *n* = 132, *p* < 0.001) and autumn (4.9% decrease; t = 7.941, *n* = 132, *p* < 0.0001) surveys. The summer decrease was not related to sex, nor to mature condition (3.47 ± 0.084, 3.20 ± 0.07 and 3.36 ± 0.10 for spring, summer, and autumn, respectively).

### 3.6. Trophic Niche

**Figure 5:**
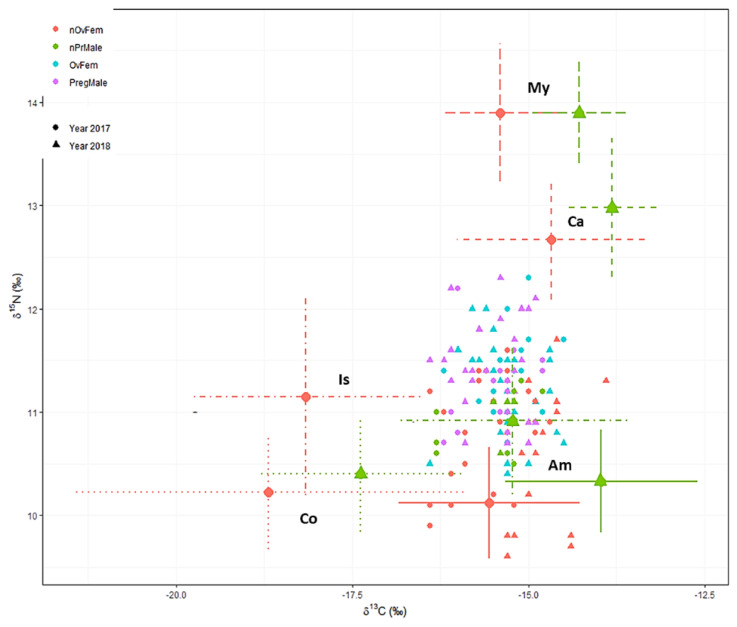
Isotopic bi-plot for reproductive stages in *Syngnathus acus* (*n* = 146; 2017 and 2018 surveys) relative to average δ^13^C and δ^15^N signatures of five potential prey sources (mean ± SD; crosses) adjusted for TDF values and consumers (colored small symbols). TDF values: 2.5 for ^13^C and 3.9 for ^15^N [58]. Two consumers with a low probability (<5%) were excluded on both the plot and the subsequent mixing model [65]. Sources: Am—Amphipoda, Co—Copepoda, Ca—Caridea, Is—Isopoda, and My—Mysidacea. Reproductive stages: nOvFem—Nonovigerous female; nPrM—Non-pregnant male; OvFem—Ovigerous female; PregMale—Pregnant male. Further information on prey isotopic data is provided in [14].

The average niche area, SEAc, in *S. acus* was 0.89, but an increase was noticed in 2018 (0.94) compared to 2017 (0.67) (Table 5). The niche area in summer (0.88) was larger than in spring (0.62) and autumn (0.71). In addition, males and individuals from site B occupied smaller niche areas. Differences across the reproductive states were mainly due to the noticeably higher area in nonovigerous females (1.19) compared to ovigerous females (0.57) and males (0.57–0.62). The results can be visualized in detail on the isotopic bi-plots in Figure 5.

**Figure 6:**
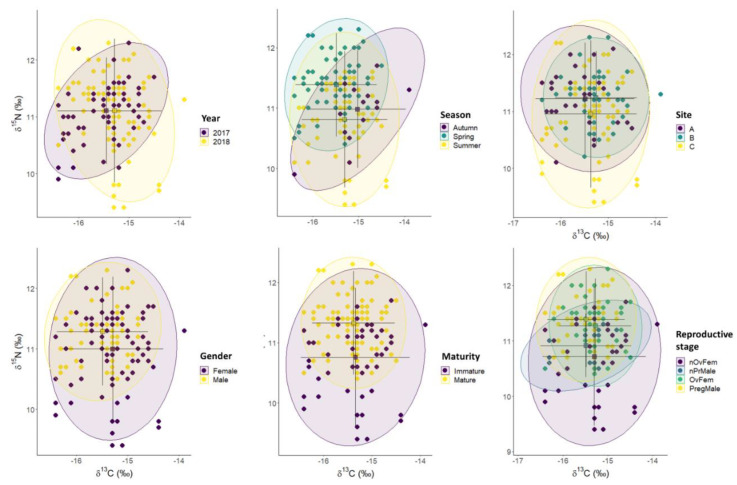
Stable isotope Bayesian ellipses showing trophic niche widths and overlaps in *Syngnathus acus* collected in spring, summer, and autumn (2017–2018), at sites A, B, and C on Cíes Archipelago. Ellipses with 95% credible intervals for the means are based on standard ellipses corrected for small sample sizes (SEAc; isotopic niche metrics; SIBER package). Each mark corresponds to the mean isotopic values. Reproductive stages: nOvFem—Nonovigerous female; nPrM—Non-pregnant male; OvFem—Ovigerous female; PregMale—Pregnant male.

Niche overlap estimates revealed a nearly full overlap of the 2017 niche by the 2018 niche, and of the spring niche by the autumn and summer niches (Table 6, Figure 6). Regarding spatial effects, the niche in individuals from site A resembled the one for site B, but the probabilities that individuals from site C occupy the niches for those in sites A and B decreased to 83.7% and 73.9%, respectively. Regarding the sexual condition, the trophic niches of males and mature fishes were markedly overlapped by those for females (95.8%) and immature fishes (93.8%), respectively. The probability that an immature female is included within the niches of non-pregnant males, pregnant males, or ovigerous females decreased to 64.8, 73.7, and 76.6%, respectively.

### 3.7. Feeding Regimes

The results of the Bayesian mixing models on the dietary regimes in *S. acus* revealed a high contribution of amphipods (21–68%) to the bulk diet in both the 2017 and 2018 surveys (Table 7, Figure S6). However, two main features deserve special consideration. The first one refers to changes in the dietary regimes across the years, with a substantial contribution of carideans (21–41%) in 2017 and harpacticoid copepods in 2018 (19–34%).

The second interesting finding refers to the changes of regimes across the four reproductive statuses recognized, with certain dissimilarities between mature and immature individuals. Accordingly, amphipods were highly consumed in nonovigerous females (46–68%) and non-pregnant males (36–63%), compared to ovigerous females (28–44%) and pregnant males (21–48%). In contrast, the contributions of copepods and isopods did not differ markedly across the reproductive states, ranging from 4 to 34% for the former, and from 3 to 24% for the latter. The low contribution of mysidaceans to the dietary regimes was noteworthy (4–20%). However, the consumption of this carnivorous component of marine zooplankton was higher in pregnant males (10–20%).

## 4. Discussion

### 4.1. Population Characteristics

The population of the great pipefish *Syngnathus acus* inhabiting Cíes Archipelago was irregularly distributed across the sites and seasons, comprising mostly very large individuals. Their occurrence extended towards less sheltered and not highly exposed zones (e.g., sites A and B). A previous study revealed that the species preferred macroalgal beds on the west coast of Cíes Archipelago [15]. As for many other syngnathids, seaweed (and seagrass) coverture play a pivotal role in the occurrence of these fishes, supplying food and shelter against predators. Environmental variables such as depth, wave exposure, and slope also govern the pattern of spatial distribution [16].

On site C, *S. acus* co-occurred sympatrically with the pipefish Entelurus aequoreus and the seahorse *Hippocampus guttulatus* [15]. Compared to the weak swimmer syngnathids such as seahorses, the great pipefish is a fast species that swims near the substrate and that may displace to adjacent areas. Undoubtedly, the high seasonal discrepancies regarding abundances and the low recapture rates would indicate a high population turnover and displacements from/to other more distant areas, especially at the onset and at the end of the breeding season. Seasonal migrations to other environments have been reported in other syngnathids aiming to avoid severe autumn–winter conditions [4,68,69,70,71].

The timings of the recaptures suggest that the half-life in the species is about one year, as previously suggested [4]. *S. acus* individuals in Cíes were markedly larger than those from nearby sites [58], Mediterranean coasts [9,10,72], the Black Sea [7], or southern Atlantic populations [73,74]. However, the large size in Cíes individuals agreed with those in eelgrass meadows populations from the northern Atlantic [4,6,75]. Hence, the length in *S. acus* adults seems to rely on its geographical origin [8]. Additionally, the adult size also seemed to determine the egg/larval size, as suggested by comparing the population from Cíes with those from the Aegean Sea [2] or from Ria de Aveiro in Portugal [13], whose eggs, larvae, and adults were notably smaller. To my knowledge, the maximal size in newborn and adult specimens from Cíes Archipelago are new records for the species.

The weight–length relationships in immature fishes (b = 3.32–3.35) agreed with the positive allometric growth reported in southern Portugal (b = 3.33–3.34) [6,76], being above and below those reported for South Africa (b = 3.07) [74] and the Aegean Sea (b = 3.71–3.73) [5,8,77], respectively. Differences in length–weight parameters across the regions might be due to dissimilarities in biotic and abiotic factors [6].

The extremely low occurrence of medium-sized specimens, the lack of juveniles, the high proportion of mature individuals, and the sharp reduction in autumnal abundances would indicate that Cíes Archipelago is an area where *S. acus* concentrates seasonally for reproduction purposes, as reported in S. schlegeli [78].

The breeding period in *S. acus* commonly extends from mid-spring to early summer [2,4], when the water temperatures are warmer. Depending on the temperature, the breeding season might also extend until late summer (2018, in the present study). In Cíes, the extension and timing of the breeding period were also governed by water temperature. In 2018, the temperatures in the summer of 2018 (18.1 ± 0.6 °C) were significantly higher than in the summer of 2017 (16.9 ± 0.2 °C), resulting in an exceptionally extended breeding season. Assuming that the duration of gestation in *S. acus* is similar to that in S. typhle (ca. 5 weeks) [79], the recapture of pregnant males in Cíes supports the existence of three annual spawning events, as in the Aegean Sea [2].

The morphology of developing embryos within the brood pouch structure was homogeneous in most broods, but genetic analyses revealed that in most cases (ca. 80%), the species follows a polygyny mating system, with a maximum of three females contributing to a single brood [38]. Females usually outnumbered males, as pointed out in Aegean Sea populations [2], but OSR were generally female-biased but seasonally variable. Hence, the unequal parental contribution [80,81] suggested in *S. acus* [4,82] should not be ruled out.

A high variability in OSR values might rely on environmental factors (e.g., temperature) that may affect the sexes differently [78,83]. The male-biased pattern observed in the advanced periods of the breeding season (i.e., summer) might be due to the temporal gap (i.e., approximately five weeks [79]) between the last egg batch releases by females and the last releases of juveniles by pregnant males. That hypothesis is in accordance with previous studies [78,84] suggesting that the pregnant males would remain sheltered on the algal bed, whereas the females might move across the patches searching for a mate or be excluded from favored habitats by intrasexual competition.

The polygyny reported in Cíes individuals is likely associated with a sex-role reversed pattern [82]. In female-biased populations, the females would compete more strongly for receptive males, whereas males would be both the more choosy sex and the limiting factor [80,85]. Sex roles in syngnathids form a continuum from conventional to reversed [86,87,88]. Intermediate sexual selection on females generally occurs in polygynous species, as in *S. acus* and in many species with the breeding structure on the tail [89]. Sexual dimorphism in the Cíes specimens seemed to be limited to an orange hue in some females. In contrast to polygynous species, female-specific ornaments as sexual signals are frequently present in polyandrous pipefishes [89,90].

### 4.2. Early Development

With the caution that the results of the present study on eggs/embryos are based on a small sample size, pregnant males and advanced stages of embryogenesis were isotopically similar. Compared to the eggs (stage B), the small degree of δ^15^N-enrichment in pre-released larvae (stages H–J) suggests a fast protein turnover in developing embryos, resulting in the excretion of lighter nitrogen and leading to ^15^N-enrichment. Additionally, the selective use of some yolk reserves such as lipids and free amino acids would occur, which is a common feature in many developing fish embryos [91,92,93,94].

In most teleosts, isotopic fluctuations in the developing embryos denote a trade-off between the egg nutrients provided by females and embryo metabolism supporting development and growth. However, syngnathids are fishes with male parental care, in which egg yolk and embryo tissues originate from both the maternal and paternal resources. The brood pouch in *S. acus* resembled that of S. abaster [95] and S. floridae [81], consisting of a ventral structure located below the tail and covered by two dermal flaps that form the pouch seal at the midline. During gestation, the chamber is filled with a mucous material that surrounds the eggs. Brooding males may supply nutrients, namely amino acids, proteins, and carbohydrates, to embryos [96,97,98]. The contribution of males to the δ^13^C and δ^15^N profiles of developing embryos is unknown, and deserves further investigation that would contribute to a better interpretation of fluctuations with the embryogenesis progress. Linking the isotopic profile in parents to those in eggs or offspring could also assist in providing information about paternal foraging without having to know the female/male, or in inferring parental isotope profiles from hatchling tissues. Isotopic correlations between mother and offspring have been reported in turtles [28,33,99] and sharks [32,100,101]. However, available information on stable isotope discrimination values between breeders-to-eggs in teleost fishes is scanty [31,58].

### 4.3. Trophic Features

The feeding regime in adult *S. acus* from Cíes agreed with other syngnathids, foraging mostly upon small crustaceans [17]. In Cíes Archipelago, the species would occupy a low trophic position among carnivorous fishes, in agreement with Jennings and van der Molen [12]. The trophic niche was highly similar to those in other co-occurring syngnathids in site C [14], with small differences across species, likely due to inter-specific dissimilarities in mouth size and shape.

In Cíes, the species preyed preferentially upon amphipods with varying proportions of other sources, namely copepods, carideans, and isopods. Since both sexes only occasionally come above the vegetal assemblage [4], the average contribution of suprabenthic mysidaceans to bulk diet was reduced, although this was more highly preferred by pregnant males, as it is less accessible to *S. acus* than epifaunal resources. The low degree of foraging on mysidaceans and the preference for amphipods agree with the gut content of specimens from the Aegean Sea and Lake Bafa [10,11]. Hence, *S. acus* is a secondary consumer, a specialist predator foraging preferentially near the substrate in vegetated habitats [4]. Despite its tiny mouth, this fish can ingest prey much larger than expected, since the prey can be cut into pieces small enough to swallow [102].

The composition of the bulk diet estimates differed across seasons, sites, sexes, and maturity states. Dissimilarities across the seasons and length classes were also reported in populations of the Aegean Sea, with more diverse feeding occurring in spring than in winter [9]. Overall, the individuals collected in summer from Cíes occupied lower trophic positions and wider niche areas (i.e., higher diversity in dietary resources), principally due to the impact of nonovigerous females. Another influential factor might be seasonal variability in epifaunal assemblages, especially from summer to autumn, resulting from changes in the macroalgal beds [14]. Gammaridae, Corophidae, and Amphilochidae are amphipods that are submitted to high seasonal variability in Cíes, especially during autumn and summer.

The trophic overlap and the SIMM results indicate dietary differences between immature specimens and pregnant males–ovigerous females, with a lower preference for amphipods in the former. Hence, mature fish seemed to be less selective than immatures, showing a higher contribution of other prey (i.e., Caridea and Copepoda, depending on the year) to bulk diet. In addition to this, the species showed certain trophic flexibility, adapting to fluctuations in prey diversity and availability across seasons and sites, independently of gender and reproduction state. The adaptive foraging capability to the changing abundances of dietary sources was in agreement with previous findings on populations from the Aegean Sea [10], and with seasonal changes reported in other syngnathids such as the pipefish S. taenionotus [69] or the seahorse H. guttulatus [19].

The wider niche area and lower trophic position in immature females were likely related to their higher relative abundance in site C, an area predominantly occupied by females. Site C is a sheltered area characterized by the occurrence of vegetal communities dominated by the fucoid Treptacantha baccata. This macroalga provides more shelter to pipefishes and other syngnathids (e.g., *Hippocampus guttulatus* and Entelurus aequoreus) in Cíes Archipelago, and higher relative abundances of Gammaridae and Corophidae than in Codium assemblages dominating sites A and B [14]. The values of δ^15^N were lower in amphipods (6.42 ± 0.28‰) compared to other potential sources (6.58–10.02‰) included in SIMMs.Consequently, a higher consumption of amphipods would explain the decrease noticed in both the δ^15^N values and the trophic position of *S. acus* immature females.

The high isotopic similarity between males and mature females would reveal long residence periods in the same habitat. In contrast, the higher isotopic variability and the larger trophic niche in immature fishes (especially females) might reflect a higher turnover (i.e., lower residence periods), resulting in higher isotopic heterogeneity. Immature females should be more active than males, occupying wider areas within the studied zone, as demonstrated in other pipefishes [103,104]. However, this hypothesis does not seem to be fully supported by the isotopic similarity in average δ^13^C values across sex and maturity condition.

The breeding pattern, the maturation condition, and the physiological processes involved in egg production and pregnancy could affect the isotopic profile in fishes. Syngnathid species differ in the use and allocation of resources for reproduction, demanding an important expenditure of energy and nutrients that can be obtained at different periods over the year [105]. The maintenance of reproductive capabilities thorough the breeding season might depend on the number of breeding events, as well as other biotic and abiotic factors. Consequently, reproductive events might determine the breeding pattern, followed by the species. Seahorses are synchronous batch spawners with a high number of annual breeding events. These fishes follow a mixed capital-income breeding strategy [106,107], where resources are gained prior to and during the breeding period. However, the annual number of breeding events in *S. acus* and other pipefishes [108] are reduced compared to seahorses. Unfortunately, the reproduction strategy in the polygamous *S. acus* has not been properly assessed, and the implications of resource availability before the breeding season on reproduction are unknown. It is feasible that *S. acus* follows a capital breeding strategy with a strong trade-off between feeding, energy storage, and further allocation to reproduction. This hypothesis would be partially supported by the lower foraging rate for amphipods and the higher δ^15^N values in ovigerous females and pregnant males, compared to immature individuals.

## 5. Conclusions

The monitoring of *Syngnathus acus* populations in Cíes Archipelago revealed marked seasonal differences in distributions and abundances, preference for shelter sites, and a breeding season that is highly associated with temperature and migratory events in late autumn. The population structure indicates that the studied area is an important reserve of extremely large breeders. The species should be considered a selective but flexible carnivorous fish that forages on amphipods as the main dietary contributor, but adapts its regime to annual and seasonal fluctuations and resource availability. However, trophic characteristics in immature fish, especially females, differed from those in breeding specimens. The former seemed to occupy a lower trophic position, a wider niche area, and a higher dietary diversity, but the factors involved in those traits need further addressing. The present study highlights the importance of assessing feeding regimes, considering both the spatio-temporal changes and reproductive status of migratory syngnathids. Inferring dietary regimes for the whole population from pooled samples taken at different locations and sampling times would lead to quantitative biases and qualitative misrepresentations of estimates. It is likely that the trophic features in the species are mainly governed both by prey availability and by migrations and local movements that are related to seasonality and maturity. Although the current threat and conservation status of *S. acus* is of least concern, the distinctive characteristics of the population inhabiting Cíes Archipelago should deserve preferential conservation actions, including habitat (macroalgal assemblages) protection/restoration, and the reduction in anthropogenic disturbances (e.g., fishing, vessel traffic and anchorage, or noise). These measures would also impact positively upon sympatric syngnathids and other co-occurring fauna, especially in sites B and C. Since knowledge on the species was highly limited, the present study provides novel information that can be useful as reference material for further studies on the same area or in other regions.

## Figures and Tables

**Table 1:** Captures and operational sex ratios (OSR) of *Syngnathus acus* specimens (total, females and males) collected in the 2017–2018 surveys on Cíes Archipelago at sites A, B, and C. Recaptured specimens are not included.

		2017				2018			
Captures	Total	A	B	C	Total	A	B	C	Total
Spring	75	9	13	13	35	14	9	17	40
Females	43	8	7	9	24	6	2	11	19
% Ovigerous	84	88	86	56	75	100	100	91	95
Males	32	1	6	4	11	8	7	6	21
% Pregnant	91	100	83	73	82	100	100	100	100
Summer	62	4	7	12	23	7	8	24	39
Females	41	2	6	9	17	7	5	12	24
% Ovigerous	29	0	0	22	12	71	40	25	42
Males	21	2	1	3	6	0	3	12	15
% Pregnant	76	50	100	100	83	-	67	75	73
Autumn	16	0	8	4	12	0	3	1	4
Females	12	0	5	3	8	0	3	1	4
% Ovigerous	0	-	0	0	0	-	0	0	0
Males	4	0	3	1	4	0	0	0	0
% Pregnant	0	-	0	0	0	0	0	0	0
Total	153	13	28	29	70	21	20	42	83
Females	96	10	18	21	49	13	10	24	47
% Ovigerous	50	70	33	33	41	85	40	54	60
Males	57	3	10	8	21	8	10	18	36
% Pregnant	79	67	60	63	62	100	90	83	89
OSR									
Spring	0.45	0.12	0.45	0.37	0.33	0.57	0.78	0.37	0.54
Summer	0.57	1.00	1.00	0.60	0.71	-	0.50	0.75	0.52
Spring + Summer	0.48	0.22	0.50	0.42	0.39	0.42	0.69	0.54	0.53

**Table 2:** Standard length (SL), wet weight (WW), isotopic profiles (δ^13^C, δ^15^N), and C:N ratios in *Syngnathus acus* males and females collected in 2017–2018 in Cíes Archipelago. Different letters indicate significant differences between groups (*p* < 0.05). Recaptured specimens were not included.

	SL (cm)	WW (g)	δ^13^C (‰)	δ^15^N (‰)
Females	33.7 ± 6.7	29.5 ± 16.6	−15.3 ± 0.5	11.0 ± 0.6
Ovigerous	36.2 ± 5.6 ^a^	34.8 ± 15.1 ^a^	−15.3 ± 0.4 ^a^	11.3 ± 0.4 ^a^
Nonovigerous	31.8 ± 6.8 ^b^	24.2 ± 16.5 ^b^	−15.3 ± 0.6 ^a^	10.7 ± 0.6 ^b^
Males	35.7 ± 6.8	39.6 ± 21.8	−15.5 ± 0.5	11.3 ± 0.5
Pregnant	36.3 ± 3.2 ^a^	41.4 ± 20.6 ^a^	−15.5 ± 0.4 ^a^	11.4 ± 0.3 ^a^
Non-pregnant	33.2 ± 8.8 ^ab^	32.6 ± 25.6 ^ab^	−15.5 ± 0.5 ^a^	10.9 ± 0.4 ^b^

**Table 3:** Standard length (SL) and isotopic values (δ^13^C and δ^15^N) in developing *Syngnathus acus* (stages B, H, I, and J, according to [43], collected from pregnant males in 2017–2018 on Cíes Archipelago. Isotopic values for pregnant males are given between the brackets. n: number of batches analyzed (20–30 specimens per batch). *: diameter (mm).

Stage	n	SL (cm)			δ^13^C (‰)				δ^15^N (‰)			
B	1	2.03	*		−16.6			(−15.1)	10.9			(12.0)
H	6	2.18	±	0.49	−16.3	±	0.5	(−15.6 ± 0.6)	11.7	±	0.4	(11.6 ± 0.3)
I	5	2.79	±	0.18	−15.2	±	0.7	(−15.6 ± 0.3)	11.8	±	0.6	(11.8 ± 0.4)
J	3	3.02	±	0.07	−16.1	±	1.2	(−16.1 ± 0.2)	11.5	±	0.4	(11.1 ± 0.4)

**Table 4:** Trophic positions (TP; mean ± sd) in *Syngnathus acus* specimens from Cíes Archipelago in 2017–2018 surveys.

	All Fish			n	Immature			n	Mature			n
Pooled	3.36	±	0.05	148	3.26	±	0.05	58	3.41	±	0.05	90
Spring	3.47	±	0.08	71	3.37	±	0.08	8	3.48	±	0.07	63
Summer	3.20	±	0.07	61	3.14	±	0.07	34	3.26	±	0.07	27
Autumn	3.36	±	0.10	16	3.36	±	0.10	16	-		-	0
Males	3.39	±	0.05	55	3.30	±	0.05	12	3.42	±	0.05	43
Spring	3.49	±	0.08	31	3.34	±	0.28	3	3.50	±	0.08	28
Summer	3.26	±	0.06	20	3.24	±	0.08	5	3.26	±	0.06	15
Autumn	3.34	±	0.17	4	3.34	±	0.17	4	-		-	0
Females	3.33	±	0.05	93	3.25	±	0.05	46	3.39	±	0.05	47
Spring	3.44	±	0.07	40	3.39	±	0.07	5	3.45	±	0.08	35
Summer	3.17	±	0.07	41	3.13	±	0.07	29	3.26	±	0.07	12
Autumn	3.36	±	0.11	12	3.36	±	0.11	12	-		-	0

**Table 5:** Estimated niche areas in *Syngnathus acus* collected in spring, summer, and autumn (2017–2018) on Cíes Archipelago. TA, SEA, and SEAc (SIBER package): Total area of convex hull, standard ellipse area, and corrected standard ellipse with a correction for small sample sizes, respectively.

	TA	SEA	SEAc		TA	SEA	SEAc
Pooled	5.60	0.88	0.89	Sex			
Year				Female	5.23	0.95	0.96
2017	3.42	0.67	0.68	Male	2.39	0.64	0.66
2018	5.17	0.94	0.95	Reproductive State			
Season				Nonovigerous female	4.32	1.16	1.19
Spring	2.80	0.61	0.62	Non-pregnant male	0.81	0.43	0.48
Summer	3.30	0.86	0.88	Ovigerous female	2.66	0.56	0.57
Autumn	2.03	0.67	0.71	Pregnant male	2.16	0.61	0.62
Site							
A	2.58	0.83	0.85				
B	2.83	0.58	0.59				
C	4.55	1.03	1.05				

**Table 6:** Niche overlap estimates (NicheROVER package) showing posterior probabilities (ɑ = 0.95) that individuals from rows will be found within the niches indicated by the column header. Results (%) are provided for years, seasons, sites, genders, maturity, and reproductive stages in *S. acus* on Cíes Archipelago (2017–2018). Seasons: Spr—Spring; Sum—Summer; Aut—Autumn. Reproductive stages (Rep Stage): nOvF—Nonovigerous female; nPrM—Non-pregnant male; OvF—Ovigerous female; PregM—Pregnant male.

Year	2017	2018	Season	Spr	Sum	Aut	Site	A	B	C	
2017	-	92.2	Spr	-	89.9	57.6	A	-	82.3	93.5	
2018	72.8	-	Sum	75.1	-	67.5	B	95.4	-	97.6	
			Aut	68.2	87.5	-	C	83.7	73.9	-	
Gender	Female	Male	Maturity	Mat	Imm		Rep Stage	nOvF	nPrM	OvF	PregM
Female	-	78.7	Mature	-	93.8		nOvF	-	64.8	76.6	73.7
Male	95.8	-	Immature	66.7	-		nPrM	99.1	-	91.6	94.3
							OvF	99.4	77.9	-	97.6
							PregM	98.2	67.8	95.6	-

**Table 7:** SIMMs (MixSIAR package)–Percentage (mean) contribution of potential prey sources to the *S. acus* diet. Values for 95% lower and upper CI are provided between brackets. Analyses based on isotopic data from 146 specimens in Cíes Archipelago (2017–2018).

	Females				Males			
Sources	Nonovigerous		Ovigerous		Non-pregnant		Pregnant	
2017								
Amphipoda ^(1)^	68	(53–80)	44	(27–61)	63	(40–80)	48	(22–68)
Caridea ^(2)^	21	(4–33)	41	(11–56)	22	(3–37)	30	(3–50)
Copepoda ^(3)^	4	(0–16)	6	(0–21)	7	(0–25)	9	(0–27)
Isopoda ^(4)^	3	(0–12)	3	(0–10)	3	(0–12)	3	(0–13)
Mysidacea ^(5)^	4	(0–15)	6	(0–25)	5	(0–17)	10	(0–28)
2018								
Amphipoda	46	(0–73)	28	(0–48)	36	(0–62)	21	(0–38)
Caridea	5	(0–15)	10	(0–30)	5	(0–19)	8	(0–34)
Copepoda	19	(10–33)	29	(1–43)	29	(10–48)	34	(1–48)
Isopoda	24	(0.81)	20	(0–61)	21	(0–70)	17	(0–58)
Mysidacea	6	(2–12)	13	(1–25)	9	(1–21)	20	(1–32)

^(1)^*Amphilochus manudens*, *Apherusa* spp., *Caprella acanthifera*, *C. linearis*, *Corophium* spp., and other gammaridae; ^(2)^ *Hippolyte varians*; ^(3)^ Harpacticoida; ^(4)^ *Cymodoce truncata*, *Dynamene bidentata*; ^(5)^ *Siriella armata*.

## Data Availability

All raw data presented in this manuscript are available at Mendeley datasets (doi: 10.17632/kf3tn3d9j8.1).

## Supplementary Materials

The following supporting information can be downloaded at: https://www.mdpi.com/article/10.3390/biology11050712/s1, **Figure S1.** Abundances in *Syngnathus acus* collected in Cíes Archipelago (2017–2018), considering reproductive states (ovigerous females, pregnant males, nonovigerous females, and non-pregnant males), seasons (spring, summer, and autumn), and sites (A, B, and C). Significances of the Kruskal–Wallis test are shown. **Figure S2.** Two-dimensional non-metric multidimensional scaling (NMDS; Bray–Curtis similarities) plot for *Syngnathus acus* individuals collected in Cíes Archipelago, considering period (years 2017 and 2018), seasons (spring, summer, and autumn), and sites (A, B, and C). The confidence limits for ellipses (95% confidence) are shown. The influence of reproductive states (ovigerous females, pregnant males, nonovigerous females, and non-pregnant males) are indicated by arrows. Each mark corresponds to mean values for duplicate samples. **Figure S3.** Relationship between wet weight and daily weight-specific growth rate in *Syngnathus acus* collected in Cíes Archipelago. Only recaptured individuals are included. **Figure S4.** Length–weight relationships in mature (continuous line) and immature (dotted line) *Syngnathus acus* males and females captured in surveys carried out in spring, summer, and autumn (2017 and 2018) on sites A, B, and C in Cíes Archipelago. **Figure S5.** SL (cm), WW (g), δ^13^C (‰), δ^15^N (‰), and C:N values in *Syngnathus acus* collected in Cíes Archipelago (2017–2018), considering reproductive states (ovigerous females, pregnant males, nonovigerous females, and non-pregnant males), seasons (spring, summer, and autumn), and sites (A, B, and C). Significances of the Kruskal–Wallis test are shown. **Figure S6:** Percentage (mean ± SD) contribution of potential prey sources to the *S. acus* diet, as estimated by the Bayesian Stable Isotope Mixing Model (SIMM) (MixSIAR package in R v. 3.1.12). Analyses based on isotopic data for the dorsal fin tissues of 146 fishes sampled on Cíes Archipelago in 2017–2018. Bayesian models run (long run; chain length = 300,000, burn = 200,000) using experimentally derived TDF values (Δ^13^C = 2.50‰; Δ^15^N = 3.91‰) (see [58] for further details). Convergence and diagnostic statistics evaluated using the Gelman–Rubin and Geweke tests. **Table S1.** Standard length (cm) and wet weight (g) in *Syngnathus acus* collected in 2017–2018 in Cíes Archipelago. Recaptured specimens are not included. **Table S2.** Isotopic profiles (δ^13^C and δ^15^N) and C:N ratios in *Syngnathus acus* collected in 2017–2018 in Cíes Archipelago. Recaptured specimens are not included.
