# Supplementary material for: Ecological Traits and Trophic Plasticity in The Greater Pipefish Syngnathus acus in the NW Iberian Peninsula
Source: Biology (Basel). 2022 May 7;11(5):712. doi: 10.3390/biology11050712 (PMC9138823; doi:10.3390/biology11050712)
Supplement: Supplementary file 1 [file biology-11-00712-s001.zip › biology-1649018-supplementary.pdf]

SUPPLEMENTARY MATERIAL

Ecological traits and trophic plasticity in the greater pipefish *Syngnathus acus* in NW Iberian Peninsula

Miquel Planas[1*]

[1] Department of Ecology and Marine Resources, Instituto de Investigaciones Marinas (CSIC), 36208 Vigo, Spain

* Corresponding author: mplanas@iim.csic.es

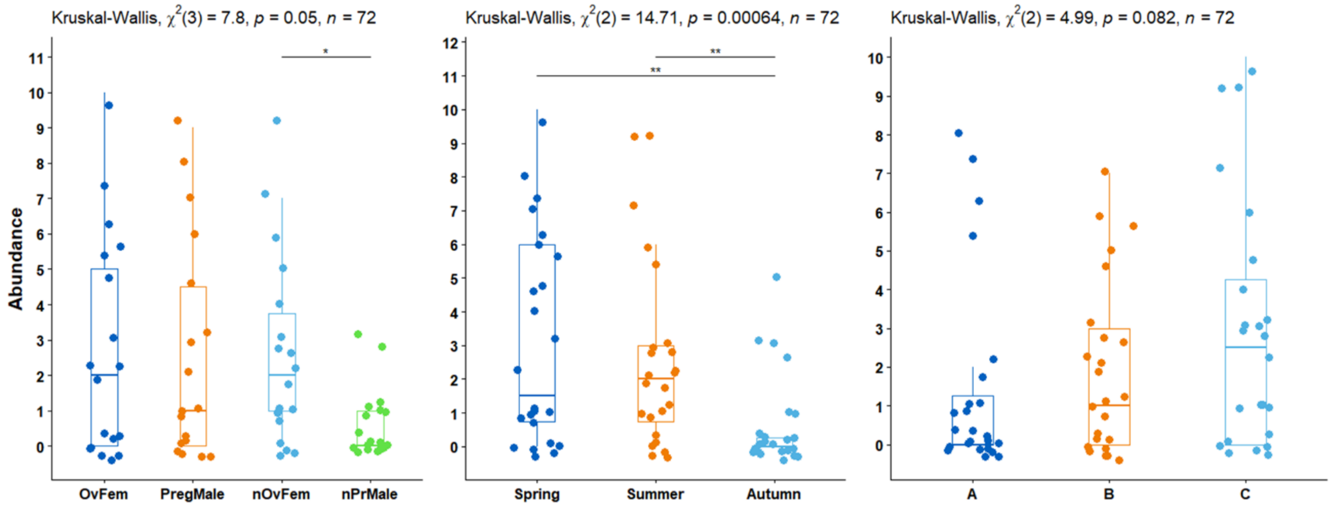

Figure S1. Abundances in *Syngnathus acus* collected in Cíes Archipelago (2017-2018) considering reproductive states (ovigerous females, pregnant males, nonovigerous females and non-pregnant males), seasons (spring, summer and autumn) and sites (A, B and C). Significances of Kruskal-Wallis test are shown.

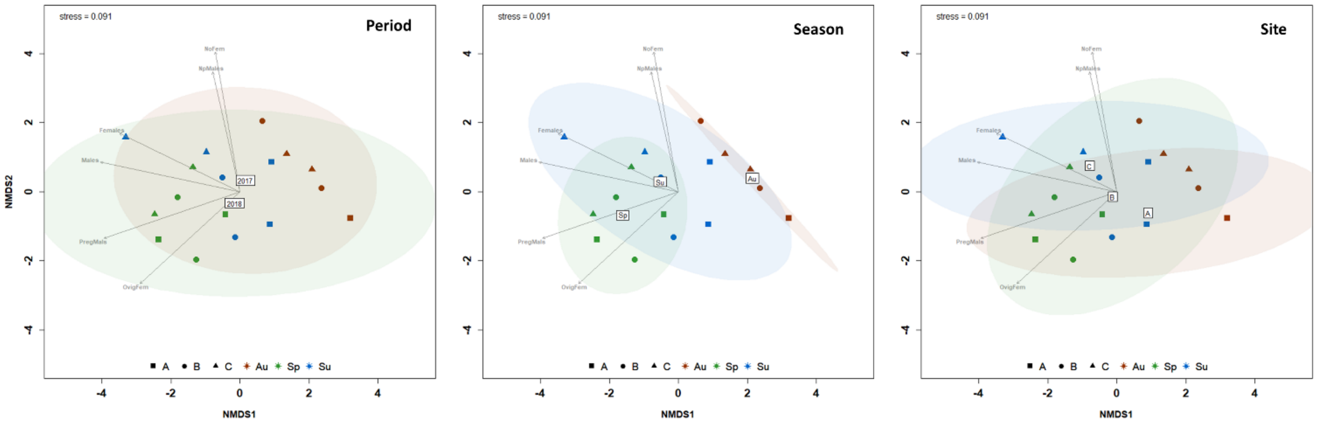

Figure S2. Two-dimensional non-metric multidimensional scaling (NMDS; Bray–Curtis similarities) plot for *Syngnathus acus* individuals collected in Cíes Archipelago considering period (years 2017 and 2018), seasons (spring, summer and autumn) and sites (A, B and C). The confidence limits for ellipses (95% confidence) are shown. The influence of reproductive states (ovigerous females, pregnant males, nonovigerous females and non-pregnant males) are indicated by arrows. Each mark corresponds to mean values for duplicate samples.

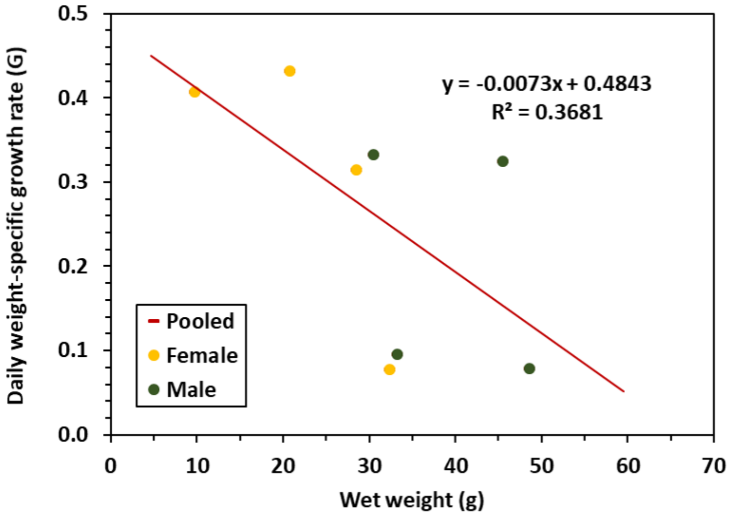

Figure S3. Relationship between wet weight and daily weight-specific growth rate in *Syngnathus acus* collected in Cíes Archipelago. Only recaptured individuals are included.

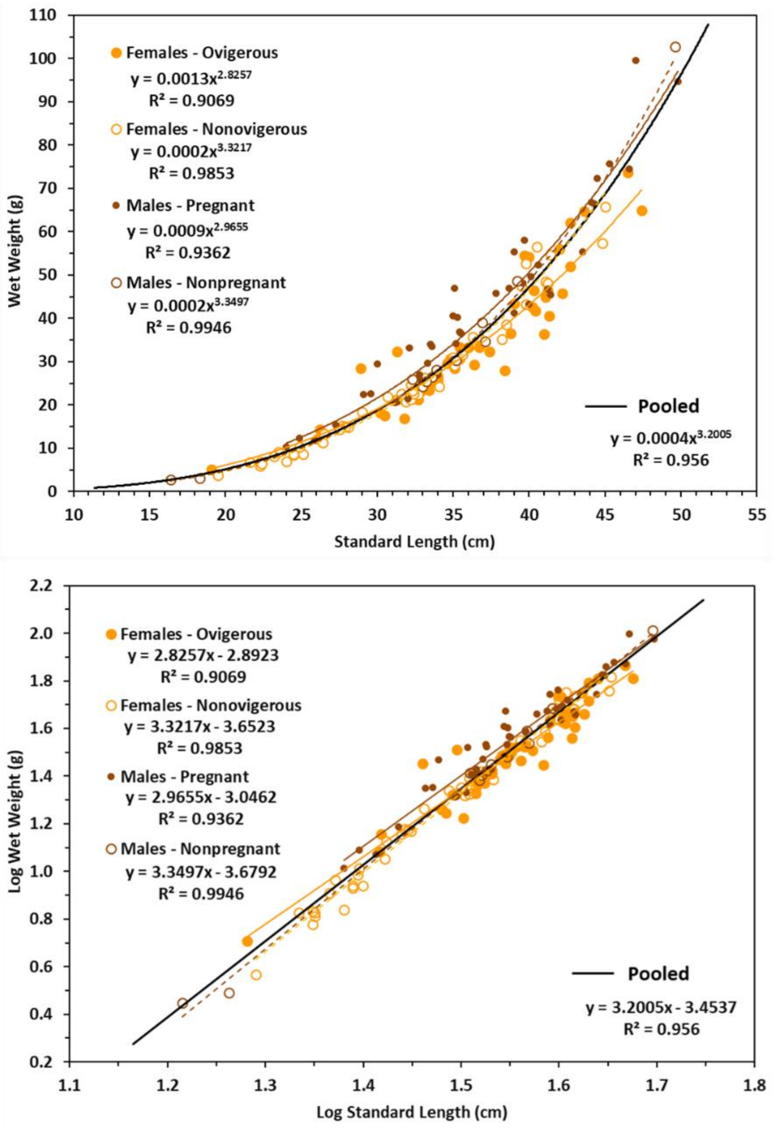

Figure S4. Length-weight relationships in mature (continuous line) and immature (dotted line) *Syngnathus acus* males and females captured in surveys carried out in spring, summer and autumn (2017 and 2018) on sites A, B and C in Cíes Archipelago.

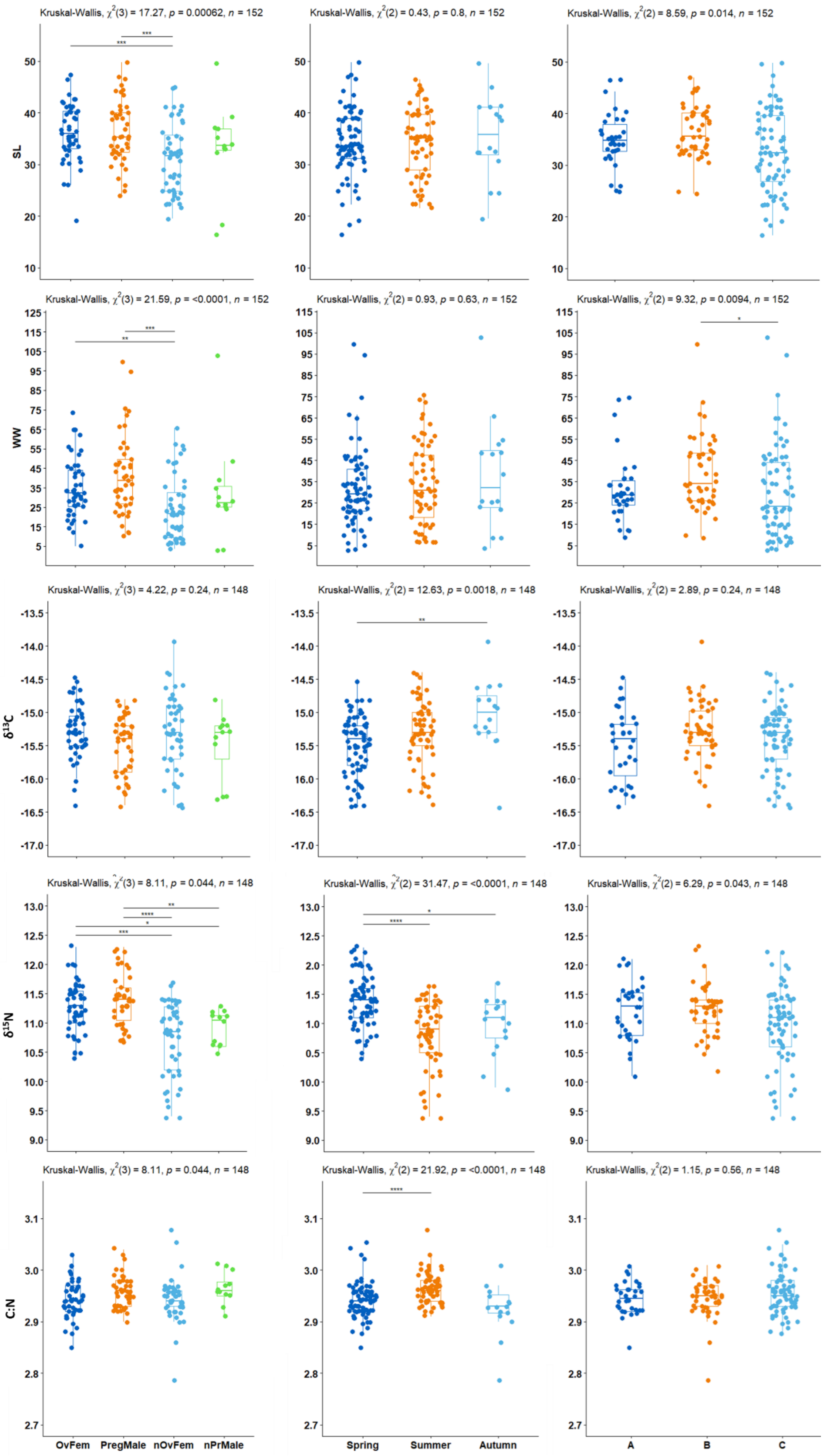

Figure S5. SL (cm), WW (g), δ^{13}C (‰), δ^{15}N (‰) and C:N values in *Syngnathus acus* collected in Cíes Archipelago (2017-2018) considering reproductive states (ovigerous females, pregnant males, nonovigerous females and non-pregnant males), seasons (spring, summer and autumn) and sites (A, B and C). Significances of Kruskal-Wallis test are shown.

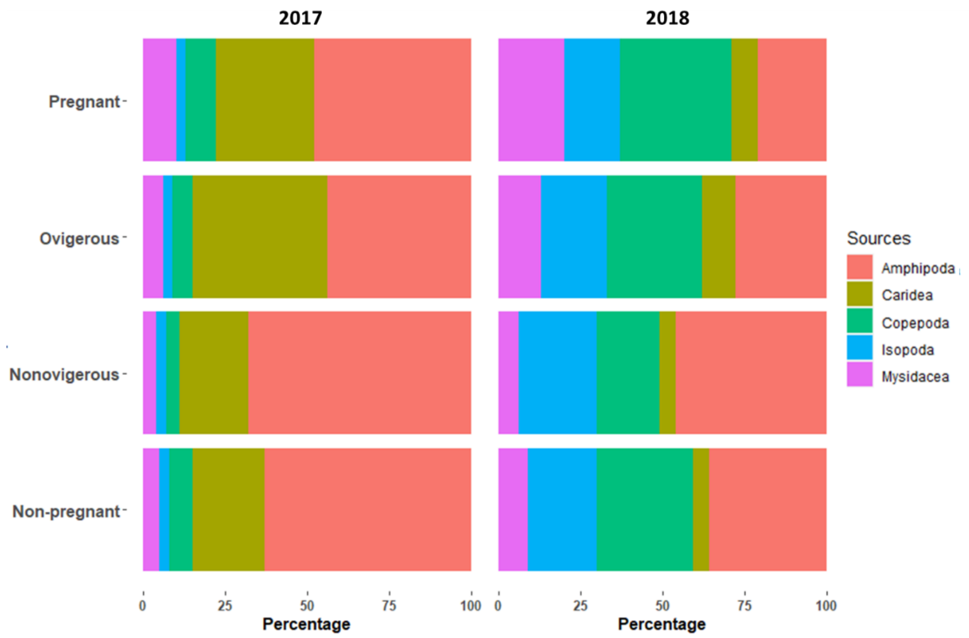

Figure S6: Percent (mean ± sd) contribution of potential prey sources to *S. acus* diet as estimated by Bayesian Stable Isotope Mixing Model (SIMM) (MixSIAR package in R v. 3.1.12). Analyses based on isotopic data for dorsal fin tissues of 146 fishes sampled on Cíes Archipelago in 2017-2018. Bayesian models run (long run; chain length=300,000, burn=200,000) using experimentally derived TDF values ($\Delta^{13}C$=2.50‰; $\Delta^{15}N$=3.91‰) (see [58] for further details). Convergence and diagnostic statistics evaluated using Gelman–Rubin and Geweke tests.

Sources: Amphipods: *Amphilochus manudens, Apherusa* spp., *Caprella acanthifera*, *C. linearis*, *Corophium* spp., and other gammaridae

Caridea: *Hippolyte varians*

Copepods: Harpacticoida

Isopoda: *Cymodoce truncata, Dynamene bidentata*

Mysidacea: *Siriella armata*.

Table S1. Standard length (cm) and wet weight (g) in *Syngnathus acus* collected in 2017-2018 in Cíes Archipelago. Recaptured specimens are not included.

	Standard length (cm)				Wet weight (g)			
	**Mean ± sd**	**Max**	**Min**	**n**	**Mean ± sd**	**Max**	**Min**	**n**
**Total**	34.4 ± 6.8	49.8	6.8	152	33.3 ± 19.3	102.7	2.8	152
**Period**								
**2017**	33.6 ± 6.9	49.6	16.4	70	30.7 ± 18.6	102.7	2.8	70
**2018**	35.2 ± 6.6	49.8	21.6	82	35.4 ± 19.7	99.6	6.0	82
**Season**								
**Spring**	34.3 ± 6.6	49.8	16.4	75	31.9 ± 18.1	99.6	2.8	75
**Summer**	34.4 ± 6.7	46.5	21.6	61	33.8 ± 19.2	75.6	6.5	61
**Autumn**	35.3 ± 8.1	49.6	19.5	16	37.7 ± 25.3	102.7	3.7	16
**Site**								
**A**	35.0 ± 5.3	46.6	24.9	34	31.5 ± 15.7	74.5	8.7	34
**B**	36.6 ± 5.0	47.0	24.5	47	39.0 ± 17.4	99.6	8.7	47
**C**	32.7 ± 8.0	49.8	16.4	71	30.3 ± 21.4	102.7	2.8	71

Table S2. Isotopic profiles ($\delta^{13}C$ and $\delta^{15}N$) and C:N ratios in *Syngnathus acus* collected in 2017-2018 in Cíes Archipelago. Recaptured specimens are not included.

	$\delta^{13}C$ (‰)				$\delta^{15}N$ (‰)				C:N			
	**Mean ± sd**	**Max**	**Min**	**n**	**Mean ± sd**	**Max**	**Min**	**n**	**Mean ± sd**	**Max**	**Min**	**n**
**Total**	-15.4 ± 0.5	-13.9	-16.4	148	11.1 ± 0.6	12.3	9.4	148	3.0 ± 0.1	3.5	2.8	148
**Period**												
**2017**	-15.4 ± 0.5	-14.5	-16.4	68	11.1 ± 0.5	12.3	9.9	68	3.0 ± 0.1	3.5	2.9	70
**2018**	-15.3 ± 0.5	-13.9	-16.4	80	11.1 ± 0.6	12.3	9.4	80	2.9 ± 0.0	3.0	2.8	80
**Season**												
**Spring**	-15.5 ± 0.4	-14.5	-16.4	71	11.4 ± 0..4	12.3	10.4	71	2.9 ± 0.0	3.0	2.9	71
**Summer**	-15.3 ± 0.5	-14.4	-16.4	61	10.8 ± 0..6	11.6	9.4	61	3.0 ± 0.0	3.1	2.9	61
**Autumn**	-15.0 ± 0.5	-13.9	-16.4	61	11.0 ± 0..5	11.7	9.9	61	3.0 ± 0.2	3.5	2.8	61
**Site**												
**A**	-15.0 ± 0.5	-14.5	-16.4	32	11.2 ± 0..5	12.1	10.1	32	2.9 ± 0.0	3.0	2.9	32
**B**	-15.3 ± 0.4	-13.9	-16.4	48	11.2 ± 0..4	12.3	10.2	48	2.9 ± 0.0	3.0	2.8	48
**C**	-15.4 ± 0.5	-14.4	-16.4	68	11.0 ± 0.7	12.2	9.4	68	3.0 ± 0.1	3.5	2.9	68
